# A new formula for predicting the actual volume of parathyroid adenoma in patients with primary hyperparathyroidism

**DOI:** 10.55730/1300-0144.5945

**Published:** 2024-12-23

**Authors:** İbrahim KILINÇ, Mustafa ORUÇ, Serap ULUSOY, Ali COŞKUN, Mehmet KILIÇ

**Affiliations:** 1 Department of General Surgery, Ankara Bilkent City Hospital, Ankara, Turkiye; 2 Department of General Surgery, Faculty of Medicine, Yıldırım Beyazıt University, Ankara, Turkiye; 3 Department of General Surgery, Faculty of Medicine, Osmangazi University, Eskişehir, Turkiye

**Keywords:** Parathyroid adenoma, tumor volume, indirect estimation techniques

## Abstract

**Background/aim:**

Knowing adenoma volume in patients with primary hyperparathyroidism is useful for surgeons during parathyroidectomy. The primary aim of our study was to identify the most accurate method for calculating adenoma volume. Our secondary aim was to determine the relationship between adenoma volume and preoperative biochemical parameters (parathyroid hormone [PTH] and calcium levels).

**Material and methods:**

The medical histories of 75 patients who underwent surgery for a single parathyroid adenoma were prospectively collected. After excision, the adenomas were placed in water-filled syringes, and their actual volume was determined according to the amount of water displaced. The radiological volume of the adenomas was calculated using the ellipsoid body formula according to the ultrasonographic dimensions. The PTH and calcium levels of the patients were retrieved from the patients’ hospital records.

**Results:**

The calculated radiological volumes correlated with the actual adenoma volumes, but there were significant differences between the two measurements. A new formula was developed based on actual volumes and radiological volumes. A positive correlation was found between the adenoma volumes and the preoperative PTH and calcium levels.

**Conclusion:**

Adenoma volume calculated using a mathematical formula based on ultrasound measurements does not accurately reflect the actual volume. Higher preoperative calcium and PTH levels are associated with larger adenoma size.

## 1. Introduction

Primary hyperparathyroidism (PHPT) is characterized by excessive parathyroid hormone (PTH) secretion, leading to hypercalcemia and hypophosphatemia. Patients with PHPT may present with nephrolithiasis, osteopenia, osteoporosis, constipation, and mental disorders. Currently, it is usually detected incidentally with a high calcium (Ca) level in laboratory results. The diagnosis is confirmed by high levels of PTH and Ca and low phosphate level [1–4]. Most patients (85%–90%) have a single parathyroid adenoma, but some may have multiple adenomas, hyperplasia, and (rarely) carcinomas [5]. Most people (85%–90%) have 4 parathyroid glands, each weighing 29.5–62.4 mg [6,7]. A normal gland is 3–6-mm-long, 2–4-mm-wide, and 0.5–2-mm-thick [8].

Excision of the adenoma(s) is the only definitive treatment for PHPT with a high success rate [9]. However, have low success rates and high morbidity [10], making it crucial for the surgeon to have an accurate estimate of the adenoma volume prior to an operation. This helps the surgeon ensure that he/she is removing the correct parathyroid tissue. Accurate estimates are also essential for achieving better cosmetic results, especially when using minimally invasive techniques [11].

Several studies have attempted to determine the relationship between biochemical parameters and adenoma volume using a mathematical formula based on the dimensions measured by ultrasonography (radiological volume) to calculate adenoma volume indirectly. However, in our study, we measured the actual adenoma volume both directly and indirectly. The primary aim of our study was to determine how accurately indirectly calculated radiological volumes reflect actual adenoma volume. The secondary aim was to determine the relationship between actual adenoma volume and preoperative biochemical parameters (PTH and Ca levels).

## 2. Material and methods

This article was prepared in accordance with the STROBE Statement.

### 2.1. General information

In our study, 75 patients with PHPT who underwent surgery for a single adenoma between 2014 and 2019 were included. Written informed consent was obtained from all patients prior to surgery. Patients with parathyroid carcinoma, parathyroid hyperplasia, multiple adenomas, or secondary or tertiary hyperparathyroidism were excluded. All operations were performed by the same two surgeons, and the ultrasonographic evaluation of each adenoma was performed using the same ultrasound device (Logic Pro 200 GE fitted with a 7.5-MHz probe; Kyunggi-Do, South Korea) by the same endocrinology specialist.

### 2.2. Biochemical parameters

The patients’ peak PTH and Ca levels, measured within 3 months before surgery, were obtained from their records.

### 2.3. Calculation of adenoma volume

**Figure 1:**
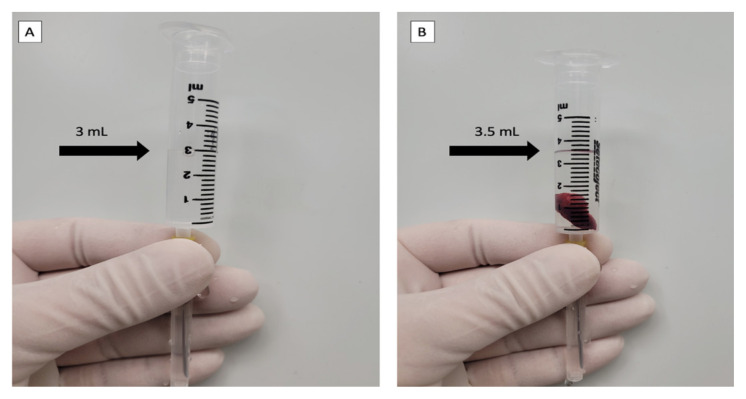
Direct measurement of adenoma volume using a water-filled syringe. A: Half-water-filled syringe; B: Water-filled syringe with adenoma. A 0.5 mm displaced water level is considered adenoma volume.

In previous studies, adenomas were evaluated as ellipsoids, and their volume was calculated using the volume formula (4/3 πabc or 4/3 πab^2^, a, b, c = radii) of the ellipsoid body. In our study, we measured adenoma volume directly by separating the adenoma from the surrounding tissues after excision and placing it in a water-filled metering syringe. The volume of the adenoma was measured by determining the amount of water displaced (Figures 1A and 1B). Before the formula was calculated, all collected values were converted from milliliters to cubic centimeters. This procedure was performed prospectively in the operating room.

### 2.4. Statistical analysis

Descriptive analysis of the patients’ characteristics and variables of interest was performed. Correlation analyses were performed using the nonparametric Spearman’s rank correlation coefficient, with actual parathyroid volume (cm3) as the dependent variable and preoperative PTH (ng/mL), Ca (mg/dL), and radiological volume as instrumental variables. Scatter plots were created to illustrate the relationships between variables. The association between radiologic volume and actual adenoma volume was investigated using linear regression modeling. The model offering the highest R-square value was selected. Before interpreting the results, assumptions for linear regression were checked using the Durbin–Watson statistic, collinearity statistics, scatter plots, and normality plots. All analyses were performed using SPSS version 20 (IBM, Armonk, NY, USA). Statistical significance was defined as p ≤ 0.05.

## 3. Results

Of the 75 patients included in our study, 58 (77.33%) were female and 17 (22.66%) were male. The mean age of the patients was 53 ± 13 years. The adenoma volumes and biochemical data of the patients are presented in Table.

**Figure 2:**
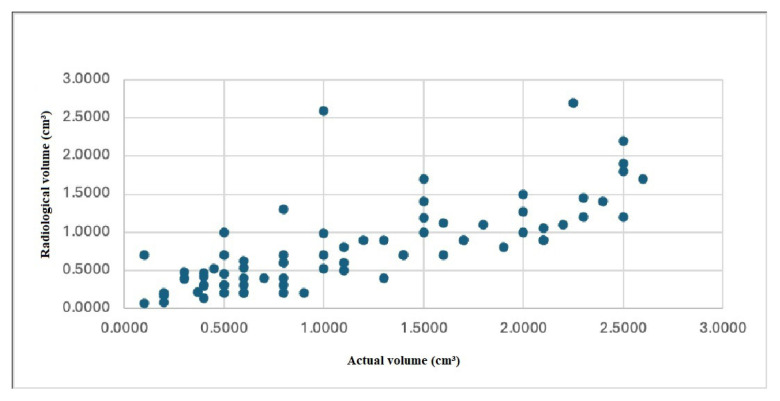
Correlation of radiological volume with the actual volume.

The mean actual volume of the adenomas was 1.1369 ± 0.740 cm3 and the mean radiological volume was 0.7924 ± 0.568 cm3. Even though there was a positive correlation between the actual volume and radiological volume (r = 0.809, p = 0.000), there was a significant difference between these two parameters (Figure 2). Based on these data, the actual volume of parathyroid adenoma was calculated with the following formula:


Actual volume=0.364+0.975×Radiological volume

**Figure 3:**
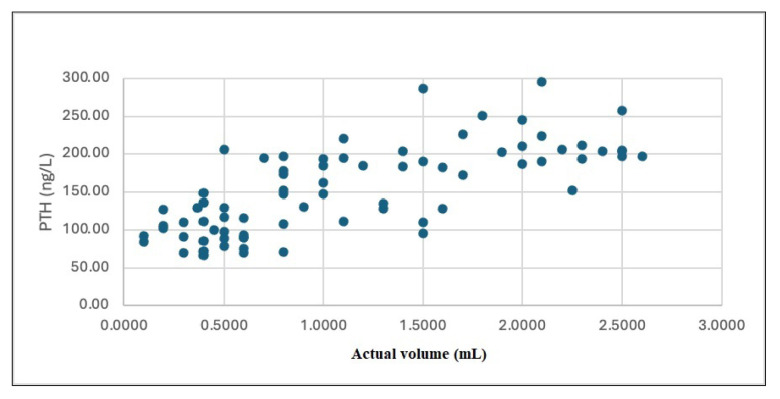
Correlation of the PTH with the actual volume. PTH: parathyroid hormone.

**Figure 4:**
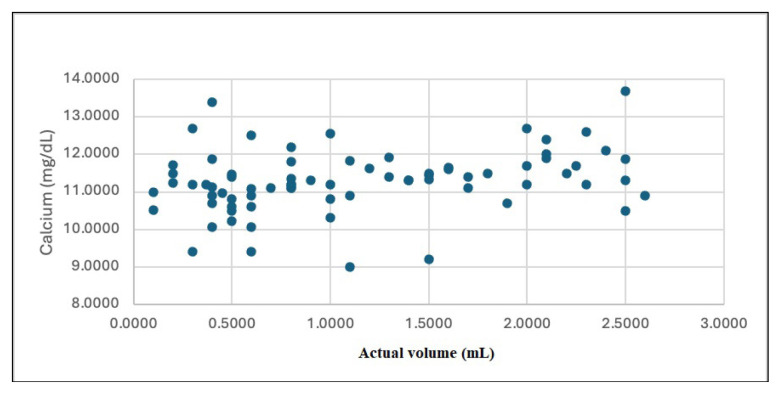
Correlation of calcium with the actual volume.

A strong correlation was found between the serum PTH level and the actual adenoma volume (r = 0.723, p = 0.001; Figure 3). There was also a correlation between serum Ca levels and the actual volume (r = 0.308, p = 0.001; Figure 4).

## 4. Discussion

In patients with PHPT, preoperative imaging methods may not always reveal the exact location of adenomas. Adenoma volume can be particularly useful in these cases [12]. Many studies have examined the relationship between biochemical parameters and adenoma volume to allow preoperative prediction of adenoma volume. In these studies, adenoma volume was calculated using mathematical formulas, but the accuracy of these estimations is not fully known. Our results indicate that such calculations do not fully and accurately reflect the actual adenoma volume; we expect that this is because adenomas are not perfectly ellipsoidal. The formula developed in the present study is a more accurate method for calculating adenoma volume during the preoperative period. We also think that using the most accurate means available to estimate adenoma volume in the preoperative period will help surgeons.

Previous studies on the relationship between preoperative biochemical parameters and adenoma volume have yielded conflicting results. Kamani et al., Bindlish et al., and Moretz et al. found a positive correlation between preoperative PTH and Ca levels and adenoma volume [11,13,14]. In their study of 52 patients, Gatu et al. found a positive relationship between PTH levels and volume, but they did not find a significant relationship between Ca levels and adenoma volume [15]. Randhava et al. found no relationship between PTH and Ca levels and adenoma weight or volume [16]. However, in our study, we found a correlation between PTH and Ca levels and adenoma volume. In studies where no correlation was found between adenoma volume and biochemical parameters, we think that this discrepancy may be due to the inability to accurately calculate adenoma volumes.

Filser et al. found a strong correlation between adenoma volume and PTH levels, as well as a positive correlation between Ca and adenoma volume. We found the same results in our study. However, this conclusion was not considered clinically important due to the weak correlation between Ca levels and adenoma volume. Filser et al. also developed a formula that includes patients’ PTH, Ca, phosphate, age, and body mass index (BMI) in the preoperative period to estimate adenoma volume:


-13.657+0.004×PTH+5.448×Ca-1.308×phosphate-0.014×age+0.041×BMI[
12]

We suggest that the formula we developed is simpler and more practical. In our study, the limited sample size of patients treated at a single center may be a potential confounding factor. Our results should be confirmed by multicenter studies with a larger number of cases.

The imaging methods currently used to calculate the volume of parathyroid adenomas do not yield accurate results. The formula presented in our study is a more accurate means of determining adenoma size in the preoperative period. Furthermore, as adenoma volume increases, PTH and calcium levels also rise.

## Figures and Tables

**Table:** Summary of patients’ adenoma volumes and preoperative biochemical parameters (n = 75).

Variables	Mean	SD	Range
Parathyroid hormone (ng/L)	153.52	56.67	67–296
Calcium (mg/dL)	11.3	1.0	9.4–13.4
Radiological volume (mm^3^)	0.792	0.568	0.070–2.700
Actual volume (mm^3^)	1.136	0.740	0.100–2.600

## References

[1] Bilezikian JP Cusano NE Khan AA Liu J-M Marcocci C Primary hyperparathyroidism Nature Reviews Disease Primers 2016 2 16033 10.1038/nrdp.2016.33 PMC5385896 27194212

[2] Fraser WD Hyperparathyroidism The Lancet 2009 374 9684 145 158 10.1016/S0140-6736(09)60507-9 19595349

[3] Khan A Hanley D Rizzoli R Bollerslev J Young J Primary hyperparathyroidism: review and recommendations on evaluation, diagnosis, and management. A Canadian and international consensus Osteoporosis International 2017 281 219 10.1007/s00198-016-3716-2 PMC5206263 27613721

[4] Walker MD Silverberg SJ Primary hyperparathyroidism Nature Reviews Endocrinology 2018 14 2 115 125 10.1038/nrendo.2017.104 PMC6037987 28885621

[5] Clark OH Duh Q-Y Primary hyperparathyroidism: a surgical perspective Endocrinology and Metabolism Clinics of North America 1989 18 3 701 714 10.1016/S0889-8529(18)30360-8 2673768

[6] Akerström G Malmaeus J Bergström R Surgical anatomy of human parathyroid glands Surgery 1984 95 1 14 21 6691181

[7] Yao K Singer FR Roth SI Sassoon A Ye C Weight of normal parathyroid glands in patients with parathyroid adenomas The Journal of Clinical Endocrinology and Metabolism 2004 89 7 3208 3213 10.1210/jc.2003-031184 15240594

[8] Harnsberger HR Osborn AG Ross J Macdonald A Diagnostic and surgical imaging anatomy: brain, head and neck, spine Salt Lake City, UT, USA Amirsys 2006

[9] Udelsman R Six hundred fifty-six consecutive explorations for primary hyperparathyroidism Annals of Surgery 2002 235 5 665 10.1097/00000658-200205000-00008 11981212 PMC1422492

[10] Sosa JA Powe NR Levine MA Udelsman R Zeiger MA Thresholds for surgery and surgical outcomes for patients with primary hyperparathyroidism: a national survey of endocrine surgeons The Journal of Clinical Endocrinology and Metabolism 1998 83 8 2658 2665 10.1210/jcem.83.8.5006 9709928

[11] Kamani F Najafi A Mohammadi S Tavassoli S Shojaei S Correlation of biochemical markers of primary hyperparathyroidism with single adenoma weight and volume Indian Journal of Surgery 2013 75 102 105 10.1007/s12262-012-0428-5 24426402 PMC3644162

[12] Filser B Uslar V Weyhe D Tabriz N Predictors of adenoma size and location in primary hyperparathyroidism Langenbeck’s Archives of Surgery 2021 406 5 1607 1614 10.1007/s00423-021-02179-9 PMC8370949 33928428

[13] Bindlish V Freeman JL Witterick IJ Asa SL Correlation of biochemical parameters with single parathyroid adenoma weight and volume Head and Neck: Journal for the Sciences and Specialties of the Head and Neck 2002 24 11 1000 1003 10.1002/hed.10165 12410535

[14] Moretz WH III Watts TL Virgin FW Jr Chin E Gourin CG Correlation of intraoperative parathyroid hormone levels with parathyroid gland size The Laryngoscope 2007 117 11 1957 1960 10.1097/MLG.0b013e31813c14fc 17891053

[15] Gatu A Velicescu C Grigorovici A Danila R Muntean V The volume of solitary parathyroid adenoma is related to preoperative PTH and 25OH-D3, but not to calcium levels Acta Endocrinologica (Bucharest) 2017 13 4 441 10.4183/aeb.2017.441 PMC6516559 31149214

[16] Randhawa P Mace A Nouraei S Stearns M Primary hyperparathyroidism: do perioperative biochemical variables correlate with parathyroid adenoma weight or volume? Clinical Otolaryngology 2007 32 3 179 184 10.1111/j.1365-2273.2007.01447.x 17550505

